# Strawberries

**Published:** 1858-04

**Authors:** 


### STRAWBERRIES.

What delightful reminiscences the word occasions! They are not here yet; but when they do come, the pleasure and the healthfulness of their free use incline us to take time by the forelock. It was estimated, last year, that *six hundred* barrels of strawberries were brought to New York daily, during the height of the season. The steamer *Keyport* alone brought on one Saturday, nine hundred and thirty-three barrels, valued at six thousand five hundred and thirty-one dollars. But it is known that the chief lusciousness of the berry is lost in large part before it gets to New York, by the jolting and pressure to which it is subjected in transportation. Were it not for that difficulty, thousands of barrels could be sent from distances not now thought of, and from country farms within fifty miles along the side of the great railroad lines; and the same may be said of the luscious peach and plum, and more or less of all other fruits. But for want of some known method of getting them to market without injury, millions—yes, millions—of bushels of fruits of all kinds go to waste, an absolute loss to the wealth of the country and to the health of the people.

We therefore do most vehemently call upon every genius in the land to let alone balloons, and aerial ships, and perpetual motions, and to expend himself in discovering something that is of some account, something that will add largely to the wealth, health, and happiness of multitudes, by devising some plan to bring berries of every description from long distances to the New York market without material compression, and as near their natural state as possible—such a man will be a blessing to humanity, and fill his purse to overflowing

